# Evidence for vitellogenin DNA‐binding in honey bees

**DOI:** 10.1002/pro.70291

**Published:** 2025-09-13

**Authors:** Gyan Harwood, Vilde Leipart, Chris Elsik, Joseph C. F. Ng, Finn Drabløs, Gro V. Amdam

**Affiliations:** ^1^ School of Life Sciences Arizona State University Tempe Arizona USA; ^2^ Faculty of Environmental Sciences and Natural Resource Management Norwegian University of Life Sciences Aas Norway; ^3^ Department of Structural and Molecular Biology University College London London UK; ^4^ College of Agriculture, Food, and Natural Resources University of Missouri Columbia Missouri USA; ^5^ School of Natural Sciences, Birkbeck University of London London UK; ^6^ Department of Clinical and Molecular Medicine Norwegian University of Science and Technology Trondheim Norway

**Keywords:** apolipoprotein B, DNA binding, gene expression, gene regulation, large lipid transfer protein superfamily, microsomal triglyceride transfer protein, vitellogenin

## Abstract

DNA‐binding proteins play essential roles in DNA replication, DNA repair, DNA organization, and several aspects of gene regulation. Their well‐studied structures and charge configurations aid in identifying similar functions in other proteins. Vitellogenin (Vg) is a highly conserved protein that is central to egg‐yolk formation in most animal taxa. The protein is largely viewed as a transporter, but effects on immunity and behavior are documented. Experiments in honey bees (*Apis mellifera*) additionally suggest a role in gene regulation. The possibility of Vg‐DNA binding has broad relevance due to Vg being phylogenetically widespread and having descendant proteins relevant to human cardiovascular health. Previously, we found that a Vg subunit can translocate to the honey bee nucleus and interact with DNA. Now, we provide a structural explanation for Vg's DNA‐binding potential by identifying conserved DNA‐binding amino acids in structural regions similar to established DNA‐binding proteins. Next, we examined how Vg‐DNA binding may elicit gene expression changes in honey bee workers, characterized by distinct changes in Vg levels over their lifetimes. Finally, we identify other nuclear proteins likely bound to the Vg‐DNA complex in honey bees. Our data suggest that Vg‐DNA binding is associated with expression changes in dozens of genes and that the Vg‐DNA complex interacts with dozens more nuclear proteins. We propose that Vg‐DNA binding can regulate several important processes in honey bee workers, including energy metabolism, behavior, and signaling. Due to the conserved nature of Vg and its descendant proteins, these functions may be present in various animals, including humans.

## 1 INTRODUCTION

Proteins' functions stem from their molecular structure, which allows them to bind specifically to and interact with various ligands, including other proteins, lipids, and nucleic acids. Many proteins, if not the majority, are multifunctional: They perform different tasks depending on a number of contexts, including their extracellular or subcellular localization, their concentration relative to binding partners, and the post‐translational modifications they have undergone (Faust et al., 2017; Gurevich & Gurevich, 2015; Volz, 2008).

Vitellogenin (Vg) is a multifunctional protein. This ancient and highly conserved protein (Babin et al., 1999; Baker, 1988a; Baker, 1988b; Smolenaars et al., 2007; Wu et al., 2013) is primarily known for its role in egg‐yolk formation in oviparous species, where it transports lipids and other nutrients into developing eggs and serves as a yolk protein precursor (Engelmann, 1979; Pan et al., 1969). In addition, Vg acts as a pathogen pattern recognition receptor (Li et al., 2008; Liu et al., 2009; Salmela et al., 2015; Tong et al., 2010; Zhang et al., 2005; Zhang et al., 2011), an antioxidant (Havukainen et al., 2013; Seehuus et al., 2006; Sun & Zhang, 2015), and a nutrient storage protein (Amdam, Norberg, et al., 2003), and plays key roles in phenotypes like behavior (Amdam, Norberg, et al., 2003; Amdam & Omholt, 2003; Dittmer et al., 2019; Ihle et al., 2010; Kohlmeier et al., 2018; Roy‐Zokan et al., 2015) and longevity (Amdam et al., 2005; Ihle et al., 2015; Salmela et al., 2016). Many of these functions are shared across disparate animal taxa, including corals (Du et al., 2017), fish (Li et al., 2008; Li et al., 2009; Liu et al., 2009; Shi et al., 2006), and insects (Harwood et al., 2019; Havukainen et al., 2013; Salmela et al., 2015).

Vg is localized both intracellularly and extracellularly, being primarily synthesized in the liver (Wang et al., 2005), adipose tissue (fat) (Brookes, 1969), or hepatopancreas (Guan et al., 2016) (depending on the organism) before being secreted into the blood or hemolymph. It can subsequently be taken up by the ovaries and other tissues via receptor‐mediated endocytosis (Koller et al., 1989; Raikhel & Dhadialla, 1992). Vg's multitude of functions has been the subject of much study, and there is now a fairly good understanding of how Vg's molecular structure enables it to interact with specific ligands to facilitate discrete functions like lipid transport (Raag et al., 1988), pathogen recognition (Liu et al., 2009; Salmela et al., 2015), and oxidative stress relief (Havukainen et al., 2013; Seehuus et al., 2006). The native structure of honey bee Vg was also recently resolved experimentally and shows how Vg can accommodate these functions (Montserrat‐Canals et al., 2025).

A less explored potential function of Vg is that of regulating gene expression. Previously, massive gene expression changes after experimental downregulation of the Vg‐encoding gene in honey bees were attributed to a co‐regulatory relationship between Vg and the insect juvenile hormone axis (Wheeler et al., 2013). Yet, more recently we found that a highly conserved structural subunit of Vg, often termed the *β*‐barrel domain, can be cleaved and translocated into the nucleus of fat body cells of honey bees, where it appears to bind to DNA at hundreds of loci (Salmela et al., 2022). We suggested that Vg might be a transcription factor or transcriptional co‐regulator, but mechanisms of Vg‐DNA binding and their consequences in honey bees or other species are so far unknown.

The Vg *β*‐barrel domain is composed of 12 *β*‐strands that fold into a nearly complete barrel. DNA binding by *β*‐strands is somewhat uncommon (Hudson & Ortlund, 2014; Yesudhas et al., 2017), but there are nonetheless examples from transcription factor families and protein domains found in a diverse range of organisms (Allen et al., 1998; Campagne et al., 2010; Cohen et al., 2003; Raumann et al., 1994; Sabogal et al., 2010; Somers & Phillips, 1992; Wojciak et al., 1999; Yamasaki et al., 2012). These include the WRKY family of transcription factors found in plants (Yamasaki et al., 2012), and the THAP zinc finger (Campagne et al., 2010; Sabogal et al., 2010) and GCM domain (Allen et al., 1998; Cohen et al., 2003) found in species ranging from bacteria to humans. In each example, DNA binding is mediated through amino acids solely on outward‐facing *β*‐strands, but they differ in how these *β*‐strands interact with DNA. In some, the *β*‐strand is aligned parallel to the DNA such that only a single side of the *β*‐strand interacts with DNA, while in others the *β*‐strand is perpendicular to the DNA—allowing interaction with both sides of the *β*‐strand. The known DNA‐*β*‐strand binding elements also have added features to support DNA interaction, such as a zinc‐binding site, a stabilizing *α*‐helix, or both. Intriguingly, the honey bee *β*‐barrel has several outward‐facing *β*‐strands, a central *α*‐helix, and two putative zinc‐binding sites (Havukainen et al., 2011; Leipart et al., 2022; Leipart, Montserrat‐Canals, et al., 2022; Montserrat‐Canals et al., 2025). Also, Vg across taxa, as well as human descendant proteins like Apolipoprotein B100, is shown to be glycosylated on several sites and consistently on *β*‐barrel domain (Clerc et al., 2016; Harazono et al., 2005; Khalaila et al., 2004; Tufail & Takeda, 2008), which has been structurally resolved on the honey bee *β*‐barrel domain (Havukainen et al., 2011; Montserrat‐Canals et al., 2025). Glycans have been demonstrated as a supporting feature for DNA binding (Kim et al., 2016).

Here, we take the understanding of Vg‐DNA binding to the next level. We start by comparing the conserved sequence and structural features of the honey bee Vg *β*‐barrel with those of the established *β*‐sheeted DNA binding proteins. Following our sequence and structural analysis, we examine how Vg‐DNA binding and gene expression changes may vary between honey bees characterized by different Vg levels due to distinct caste formation in their social division of labor. This approach is possible because Vg affects worker behavioral development, with younger workers aged 1–2 weeks having high titers of Vg and performing brood‐rearing tasks (i.e., nursing), while a subsequent, naturally occurring or induced decline in Vg and a concomitant increase in juvenile hormone prompt workers to become foragers that collect resources required by the colony ((Amdam & Omholt, 2003)—and as validated by RNA interference‐mediated knockdown of *vg* gene activity (Amdam, Simões, et al., 2003; Nelson et al., 2007)). In this study, we control age (a natural confounder of worker behavior) by comparing age‐matched nurses and foragers sampled from single‐cohort colonies (see Methods) before mapping out Vg‐DNA binding sites and closeness to promoter regions using chromatin immunoprecipitation followed by sequencing (ChIP‐seq). Next, we use RNA‐seq to study how Vg‐DNA binding corresponds to gene expression differences, and we perform gene ontology (GO) term analyses to assess our data for enrichment of specific biological functions. Finally, we use co‐immunoprecipitation followed by mass spectrometry to identify additional nuclear proteins that are likely bound to the Vg‐DNA complex.

Our sequence and structural analysis show that honey bee Vg has functional sites in highly relevant protein regions, supporting the feasibility of direct DNA‐binding by its *β*‐barrel domain. Based on our cumulative findings from ChIP‐seq, RNA‐seq, and co‐immunoprecipitation, moreover, we speculate that Vg‐DNA binding can regulate many genes involved in energy metabolism, behavior, and signal transduction pathways. Taken together, our results represent the first comprehensive investigation of the potential for direct gene regulation by Vg. Given Vg's ubiquity in metazoans and its conserved sequence and structure, we hope that our work will inspire new research in a variety of organisms.

## 2 METHODS

### 2.1 Sequence analysis

We used a transformer‐based meta‐predictor hybridDBRpred (Zhang et al., 2024), which combines predictions generated by four different tools, to identify predicted DNA‐binding amino acids in the *β*‐barrel domain of honey bee Vg (data input: amino acid sequence 20–323 from UniProt ID: Q868N5). To identify conserved residues important for the structure and function of the *β*‐barrel, we searched UniProt with a strict threshold (>*e* ^−20^) likely to identify homologs of similar structure or function. The *β*‐barrel sequence, same as above, was queried in Jackhmmer (3 iterations, v.2021_04) (Potter et al., 2018) to identify all relevant sequences (2733 sequences) and applied a 20% length filter to only include proteins with similar lengths. Next, we used FunFHMMER (Sillitoe et al., 2015) to classify the sequences into functional families, where one grouping represents protein domains likely to have the same function (Das et al., 2015). Two iterations resulted in 53 functional families. The functional family of honey bee Vg included 261 sequences and had a high diversity score (DOPS) of 0.873 (with a range of 0–1). A DOPS score >0.80 means the multiple sequence alignment (MSA) has high diversity, and the highly conserved residues will likely have a functional role (Das et al., 2015). We calculated the conservation score of residues by using ScoreCons (Valdar, 2002), and considered amino acids conserved when reaching a score of >= 0.70. We identified DNA‐binding amino acids in human microsomal triglyceride transfer protein (MTP) and Apolipoprotein B100 (ApoB) using hybridDBRpred (data input for MTP: amino acid sequence 29–273 from UniProt ID: P55157, and data input for ApoB: amino acid sequence 46–298 from UniProt ID: P04114). We used the alignments from CATH superfamily 2.30.230.10 (Orengo et al., 1997), specifically functional families 1 (MTP) and 3 (ApoB), to identify conserved residues. The results are presented in Supplement Figure S2

### 2.2 Structural analysis

We used three structural alignment tools: TM‐align (Zhang & Skolnick, 2005), FoldMason (Gilchrist et al., 2024), and SSAP (Orengo & Taylor, 1996), which all use different features: TM‐align compares the structures directly to obtain the best overall global alignment. FoldMason's scope is similar to TM‐align, but instead uses a structural alphabet (3Di). SSAP, on the other hand, uses the spatial relationship between residues to compare structures. Each method provides different outputs. TM‐align calculates the root mean square deviation (RMSD, the distance between two corresponding atoms between two proteins) and TM‐score (0–1, where 0.0–0.3 indicates random structural similarity and >0.5 indicates a similar fold), in addition to sequence identity. FoldMason calculates local distance difference test (LDDT from 1 to 0, where 1 is maximum similarity). SSAP calculates a SSAP score (0–100, where a score >80 indicates a highly similar structure, while >60 indicates common structural motifs), in addition to the number of aligned residues, percentage of overlap between the structures, sequence identity, and RMSD. Using this battery of assessment tools, we aligned the honey bee *β*‐barrel experimental resolved structure (Leipart et al., 2025; Montserrat‐Canals et al., 2025) to all experimental structures available from the three families of *β*‐sheeted DNA binding domains, including zinc‐binding sites: WRKY, GCM, and THAP zinc fingers. The Protein Data Bank (PDB) IDs are 5w3x, 2ayd, 2lex, 1wj2, 1odh, 3kde, 2jm3, and 2lau. Figures for visualization were created using PyMol (Schrödinger, n.d.) and ProteoVision (Penev et al., 2021).

### 2.3 Bees

Bee stocks were maintained at the Arizona State University Bee Research Facility in Mesa, Arizona, USA. Insect studies do not require board approval, but all bees in this study were handled and euthanized humanely. We established 3 single‐cohort colonies and treated each as a separate biological replicate owing to the high degree of relatedness among the nestmates. We followed established procedures as single‐cohort colonies are a gold standard for controlling age in experiments with worker honey bees (e.g., Whitfield et al., 2003). In brief: Brood frames from established hives were placed in an incubator overnight at 34°C and 50% humidity, and the next morning roughly 2000–3000 newly emerged workers were transferred into each of 3 nucleus (small) hives along with a caged queen and frames of honey, pollen, and empty comb. The bees in each of these single‐cohort colonies originated from different donor hives. The nucleus hives were sealed for 3 days to consolidate, after which they were opened, and the queen was released from her cage. In single cohort colonies, some of the workers will prematurely transition into foragers in order to collect resources for the colony, and on the 7th day after establishing the colonies we paint marked new foragers that were returning to the hive with pollen. On the 14th day, we collected *N* = 50 paint marked foragers and an equal number of nurses from each colony, meaning all foragers and nurses had been performing their given task for at least 7 days. Nurses were identified when they entered brood cells to feed larvae. Collected bees were anesthetized on ice before having their fat body dissected, flash frozen in liquid nitrogen, and stored in −80°C. From each of the three single‐cohort colonies, we pooled fat body samples from 25 nurses or 25 foragers. Pooled samples were homogenized in a mortar and pestle with liquid nitrogen, and the resulting homogenate was divided into three workflows for ChIP‐seq, RNA‐seq, and co‐immunoprecipitation with mass spectrometry.

### 2.4 Chromatin immunoprecipitation

For the ChIP‐seq pipeline, we followed previously established protocols (Bai et al., 2013). Briefly, the homogenate was transferred to a 15 mL dounce homogenizer tube on ice with 4 mL of 1% formaldehyde in 1× phosphate‐buffered saline (PBS) to crosslink DNA and proteins in the sample. The crosslinking was quenched after 20 min by adding glycine (final concentration 125 mM). The homogenate was transferred to a 15 mL tube and centrifuged at 1500*g* for 3 min at 4°C, and the resulting pellet was washed 3 times with 1× PBS and protease inhibitor cocktail (Roche cOmplete™). We then washed the pellet once with cell lysis buffer (NaCl 100 mM, HEPES [pH 7.6] 5 mM, EDTA 1 mM, NP‐40 0.5%) and protease inhibitor and centrifuged it at 1500*g* for 5 min at 4°C. After discarding the supernatant, we resuspended the pellet in 600 μL nuclear lysis buffer (HEPES [pH 7.6] 50 mM, EDTA 10 mM, Na‐deoxycholate 0.1%, N‐lauroylcosanine 0.5%) and sonicated the sample with a QSonica Q800R2 sonicator for a total of 5 min (15 s on, 20 s off, amplitude = 20%). We checked the sonicated sample on an agarose gel to confirm that the DNA was sheared to a size of 300–500 bp.

The Vg‐DNA complex was precipitated using antibodies specific to the whole honey bee Vg protein (Pacific Immunology, Ramona, CA) and whose specificity to Vg has been verified in several studies (Harwood et al., 2019; Münch et al., 2015). We first conjugated these antibodies to magnetic Dynabeads™ (Invitrogen™ #10001D, Protein A). To do so, we removed the magnetic beads from their buffer and washed them 4 times with 1 mL blocking buffer (1× PBS with 5% bovine serum albumin [BSA, Jackson ImmunoResearch #001‐000‐161]) before adding 500 μL block solution and 20 μL antibodies to the beads and rotating overnight at 4°C. We then washed the antibody‐bead complex 5 times with blocking buffer, then added 500 μL of the chromatin extracts and 500 μL of dilution buffer with protease inhibitor (SDS 0.01%, Triton x100 1%, EDTA 1.2 mM, Tris–HCl [pH 8.0] 16.7 mM, NaCl 167 mM) and rotated at 4°C overnight. Separately, we used 50 μL of chromatin extracts + dilution buffer as *input DNA*. These samples were incubated with magnetic beads that had undergone the same washing steps, except they had not been conjugated with the antibodies. The next day, all samples were washed with a low‐salt buffer (SDS 0.01%, Triton x100 1%, EDTA 2 mM, Tris–HCl [pH 8.0] 20 mM, NaCl 150 mM), a high‐salt buffer (SDS 0.01%, Triton x100 1%, EDTA 2 mM, Tris–HCl [pH 8.0] 20 mM, NaCl 500 mM), a LiCl buffer (LiCl 0.25 M, NP40 1%, Na‐deoxycholate 1%, EDTA 1 mM, Tris–HCl [pH 8.0] 10 mM), and TE buffer (EDTA 1 mM, Tris–HCl 10 mM). The samples were then eluted by suspending the beads in 200 μL elution buffer (EDTA 10 mM, Tris–HCl [pH 8.0] 50 mM, SDS 1%) and heating in a water bath at 65°C for 15 min. The magnetic beads were discarded, and the remaining solution was reverse cross‐linked by incubating at 65°C overnight on a heating rack. To purify the DNA in the samples, we added 200 μL of TE buffer and 8 μL of RNase A (LifeTech #12091021) and incubated at 37°C for 30 min before adding 8 μL of Proteinase K (LifeTech #25530049) and incubating at 55°C for 1 h. We then used phenol:chloroform:isoamyl (LifeTech #15593‐031) and a heavy phase lock gel tube (5 PRIME #2302810) to separate the aqueous DNA from any contaminants. Finally, we used 16 μL glycogen (LifeTech #AM9510), 40 μL of sodium acetate (LifeTech #R1181), and 800 μL of 100% EtOH (Fisher #04355222) to precipitate the DNA for 1 h at −80°C before centrifuging at 16,000*g* for 30 min at 4°C. We washed the resulting pellet with 70% EtOH and allowed it to airdry before resuspending it with 50 μL of TE buffer. We measured the concentration of DNA on an Invitrogen Qubit™ Fluorometer 2.0.

### 2.5 DNA library prep, sequencing, and annotation

DNA samples (ChIP DNA + Input DNA) were submitted to the Biodesign DNASU Sequencing Core at Arizona State University. Illumina compatible libraries were generated on the Apollo 384 liquid handler using KAPA Biosystem's LTP library preparation kit (KAPA KK8232). Genomic DNA was sheared to approximately 400–600 bp fragments using Covaris M220 ultrasonicator, then all samples were end repaired and A‐tailed as described in the KAPA protocol. Illumina‐compatible adapters with unique indexes (IDT #00989130v2) were ligated on each sample individually. The adapter ligated molecules were cleaned using Kapa pure beads (Kapa Biosciences, KK8002), and amplified with Kapa's HIFI enzyme (KK2502). Each library was then analyzed for fragment size on an Agilent's Tapestation, and quantified by qPCR (KAPA Library Quantification Kit, KK4835) on Thermo Fisher Scientific's Quantstudio 5. Libraries were then multiplexed and sequenced on 2 × 75 flow cell on the NextSeq500 platform (Illumina) at the ASU's Genomics Core facility.

The raw Illumina 2 × 75 bp pair‐end reads were quality checked using FastQC v0.10.1, followed by adapter trimming and quality clipping by Trimmomatic 0.35. Any reads with start, end, or the average quality within a 4 bp window falling below quality scores of 18 were trimmed. The clean reads were aligned to the reference genome *Apis mellifera* Amel_HAv3.1 (https://www.ncbi.nlm.nih.gov/assembly/GCF_003254395.2/) by Bowtie 2 version 2.2.9. Library insert size was checked by Picard Tool (https://broadinstitute.github.io/picard/). Library complexity was checked by nonredundancy fraction (NRF), defined as the number of unique start positions of uniquely mappable reads divided by the number of uniquely mappable reads. IGVtools and bamCompare from deepTools were employed to compare two BAM files based on the number of mapped reads. First, the genome is partitioned into bins of equal size, and then the number of reads in each bin is counted. The log2 value for the ratio of the number of reads per bin of each sample was reported for IGV visualization and compared between each pair. With 95% correlation, three biological replicates were combined for peak identification. MACS2 was used for peak calling with a 0.05 false discovery rate (FDR) cutoff.

Narrowpeak files as MACS2 output for each individual replicate, and combined samples were annotated by HOMER. It first determines the distance to the nearest transcription start site (TSS) and assigns the peak to that gene. Then it determines the genomic annotation of the region covered by the center of the peak, including promoter (1 kb upstream to 100 bp downstream of TSS), transcription termination site (TTS), exon (Coding), 5′ UTR exon, 3′ UTR exon, intronic, or intergenic.

### 2.6 Genome region analysis of ChIP‐seq binding sites

To determine whether Vg‐DNA binding sites are more likely than chance to be located in promoter regions, and which sites are shared or unique to nurses and foragers, we first created a null distribution of ChIP loci. To do this, we took 1000 hypothetical ChIP peaks of 200 bp long and randomly distributed them throughout the genome using HOMER, and then repeated this procedure for 1000 iterations. We then examined which types of genomic regions were at the center of these peaks using the same default classifications in HOMER as above. A Chi‐square test was then used to determine whether to compare this null distribution with the distributions found in our ChIP‐seq data.

### 2.7 RNA extraction

Using the same homogenized samples as with the ChIP procedure, a portion of the homogenate went into an RNA extraction workflow. Here, we followed standard phenol RNA extraction protocols using TRIzol™ Reagent (Invitrogen™ #15596026), chloroform (Alfa Aesar #32614) and isopropanol (LabChem #LC157501). The resulting RNA pellets were air‐dried, re‐suspended with nuclease‐free water, and subjected to DNAse treatment (Invitrogen™ Turbo DNA‐free™ #AM1907). We measured the RNA concentration using a NanoDrop and then diluted each sample to a working concentration of 200 ng/μL.

### 2.8 RNA library prep, sequencing, and annotation

Using KAPA's mRNA HyperPrep Kit (KAPA #KK8580), mRNA sequencing libraries were generated from total RNA. Magnetic oligo‐dT beads were used to capture mRNA specifically, and the mRNA was sheared to approximately 150–200 bp in length using heat and magnesium. The first strand of the mRNA fragments was reverse transcribed using random priming. The second strand was generated with incorporated dUTP molecules, and dAMP was added to the 3′ ends of the double‐stranded cDNA molecules. Illumina‐compatible adapters with unique indexes (IDT #00989130v2) were ligated on each sample individually. The adapter ligated molecules were cleaned using KAPA Pure beads (KAPA #KK8002) and amplified with Kapa's HIFI enzyme (KAPA KK2502). The strand marked with dUTP is not amplified, allowing for strand‐specificity. Each library was then analyzed for fragment size on an Agilent Tapestation, and quantified by qPCR (KAPA KK4835) on Thermo Fisher Scientific's Quantstudio 5 before multiplex pooling and sequencing a 1 × 75 flow cell on the NextSeq500 platform (Illumina) at the ASU Genomics Core facility.

The *A. mellifera* transcriptome and Annotation Release 104 from NCBI (derived from genome Amel_HAv3.1) were used for quasi‐mapping and count generation. FASTQC1 (version 0.11.8) was used on each sample for quality check. Average per‐base read quality scores were over 30 in all samples and no adapter sequences were found, indicating high quality reads. We used Salmon3 version 0.13.1 to quasi‐map reads to the transcriptome and quantify abundance of each transcript. The transcriptome was first indexed, then quasi‐mapping was performed to map reads to transcriptome using additional arguments ‐‐seqBias and ‐‐gcBias to correct sequence‐specific and GC content biases and ‐‐numBootstraps = 30 to compute bootstrap transcript abundance estimates. Gene‐level counts were then estimated based on transcript‐level counts using the “bias corrected counts without an offset” method from the tximport package. 84%–90% of reads mapped to the transcriptome (41.1–62.2 million per sample) and were kept for statistical analysis. We used the trimmed mean of M values (TMM) normalization in the edgeR package to adjust for possible biased in RNA composition, such as reads mapping to viral genomes. Normalization factors ranged from 0.52 and 1.35, but variation was between individuals, not caste or hives, suggesting no group‐level difference in RNA composition. Samples with a higher proportion of reads mapping to viral genes tended to have lower TMM normalization factors, to account for the smaller number of reads mapping to the *A. mellifera* transcriptome. The NCBI Amel_v3.1 Annotation Release 104 contains 12,090 genes, but not all are expressed in our samples at detectable levels. We set a detection threshold of 0.5 CPM (counts per million) in each sample, resulting in 9134 genes detected that accounted for 99.95% of all reads. After filtering, TMM normalization was performed again and normalized log2‐based CPM values were calculated using edgeR's cpm() function with prior.count = 3 to help stabilize fold‐changes of extremely low expression genes. Multidimensional scaling in the limma7 package was used to identify potential treatment effects at higher level. Testing for differentially expressed genes (DEGs) was performed using Limma‐trend methods. We identified 468 DEGs between nurses and foragers using a (Benjamini–Hochberg [BH]) FDR <0.05 as a cutoff.

### 2.9 Protein immunoprecipitation and mass spectrometry

Given that transcriptional regulation can involve a complex interaction between transcription factors, co‐regulators, enzymes, signaling molecules (Bondos & Tan, 2001), and so forth, the aim here is to elucidate regulatory pathways that may contribute to Vg‐DNA binding and subsequent gene expression changes. To pull down other proteins that are interacting with Vg and DNA, we integrated our ChIP procedure with a protocol for immunoprecipitation of chromatin‐interacting protein complexes (Bailey, 2011). As with the ChIP protocol, homogenate from 25 pooled bee fat bodies from each worker caste was crosslinked with 1% PFA. We quenched the homogenate, washed it, lysed the cellular membranes, lysed the nuclear membranes, and then sheared the samples via sonication. We bound our anti‐Vg antibodies (20 μL) to 100 μL of magnetic Dynabeads™ (Protein A) then incubated them with our samples. As a control, we incubated samples with Dynabeads™ that had not been bound with anti‐Vg antibodies, so any proteins precipitated in these samples would be due to proteins interacting directly with the Dynabeads™. The remainder of the procedure followed Mohammed et al.'s protocol (Bailey, 2011), whereby the bead samples were washed first with RIPA buffer (HEPES [pH 7.6] 50 mM, EDTA 1 mM, Na‐deoxycholate 0.7%, NP‐40 1%, and LiCl 0.5 M) then with ammonium bicarbonate, then subjected to trypsin digestion, solid phase extraction, and washing, before being loaded onto an AB Sciex 4800 mass spectrometer for MALDI‐TOF/TOF‐MS (matrix‐assisted laser desorption/ionization—tandem time of flight—mass spectrometry).

Proteomics analysis was performed on an Orbitrap Fusion Lumos Tribrid mass spectrometer with an Ultimate 3000 nano‐LC and nanoelectrospray ionization. Peptides were separated with a nC18 analytical column (C18 Pepmap 100, 3 μm particle, 100 Å pore, 75 μm i.d. ×150 mm) using 150 min buffer gradient a low flow rate at 300 nL/min. Data‐dependent acquisition in positive mode was performed for data collection. Acquired data was searched with Proteome Discoverer 2.2 using the SEQUEST search engine with label‐free quantification workflow against the UniProt database of *A. mellifera* (http://www.uniprot.org; Proteome ID: UP000005203). Search parameters were trypsin cleavage sites with a two missed cleavage site allowance, precursor and fragment mass tolerance was set at ±10 ppm and 0.6 Da. Carbamidomethyl of cysteine was set as a fixed modification, and oxidation of methionine as a variable modification. Protein abundance levels were normalized by the total number of peptides analyzed per precipitations prior to generating visualization. To prioritize for potential Vg‐interacting proteins, we removed peptides which were not mapped to *A. mellifera* proteins, and performed differential abundance analysis comparing the control and treatment (i.e., anti‐Vg+) precipitations using the DESeq2 package (v.1.32.0), filtering for proteins enriched in the treatment condition with FDR <0.05. We attempted to annotate uncharacterized proteins by querying their sequences against the ClusteredNR database (accessed July 11, 2025) using the NCBI BLAST webtool (Altschul et al., 1990).

### 2.10 Gene ontology

Gene lists from ChIP‐seq and RNA‐seq were searched for enrichment of specific GO terms with HymenopteraMine (v1.4) (Mohammed et al., 2016) using Official Gene Set 3.2 (OGSv3.2) as the background. Specifically, we looked for enrichment of biological processes, molecular functions, and KEGG pathways. We used a BH‐adjusted *p* <0.05 to determine significantly enriched terms. In our analysis, we only included genes or proteins that were shared by at least 2 of the 3 biological replicates from each worker caste.

## 3 RESULTS

### 3.1 Vg DNA binding site

**FIGURE 1:**
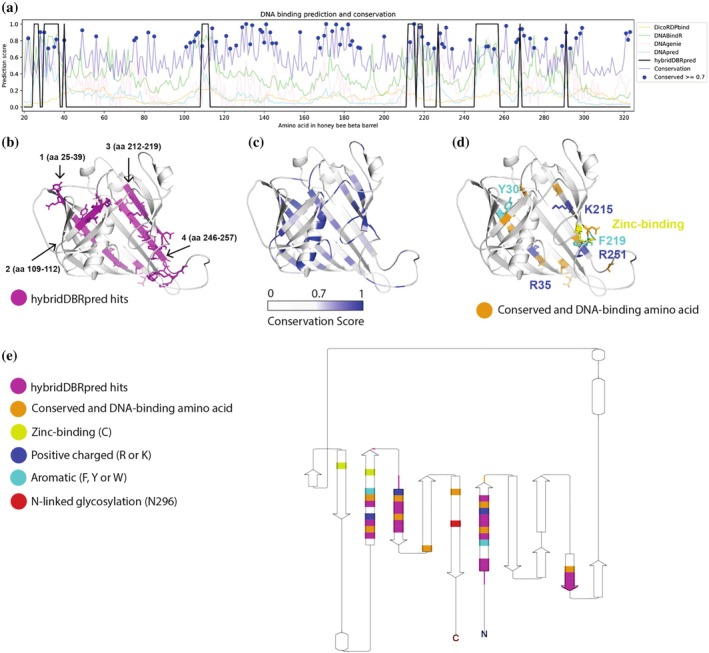
(a) HybridDBRpred combines DNA prediction scores (*y*‐axis) from four different tools (saturated yellow, green, pink, and cyan lines) that are combined into a binary DNA‐binding prediction (1 yes or 0 no, black line) for all amino acids in the *β*‐barrel (*x*‐axis). In the same plot, we have included the conservations score (blue line) and labeled conserved amino acids >= 0.7 with spheres. (b) The DNA‐binding predicted amino acids (magenta), and the 4 *β*‐strands are labeled on the *β*‐barrel protein structure (light gray). (c) Same structure as in panel (b), but colored by conservation score (from 0.7 to 1 in blue scale). (d) Same structure as in panels (b) and (c), but amino acids that are both conserved and predicted to be DNA‐binding are colored orange. Of these, the aromatic amino acids are colored cyan, the positively charged are colored blue, and the C178 and C222 predicted to be involved in zinc‐binding are colored yellow. (e) A simplified 2D map of the *β*‐barrel colored by hybridDBRpred hits (magenta), if overlapping with conserved amino acid they are colored orange, putative zinc‐binding cysteines are yellow, positive amino acids are blue and aromatic amino acid are cyan, and the N296 glycosylation site are red.

We found 38 predicted DNA‐binding amino acids in the Vg *β*‐barrel (Black line in Figure 1a). Thirty‐four of the 38 predicted DNA‐binding amino acids are located on 4 *β*‐strands (Figure 1b). By building a functional family for the Vg *β*‐barrel (see Methods for details), we identify that 15 out of the 38 sites are conserved (the calculated conservation score is from 0 to 1, where 1 is maximum conservation and the threshold for conserved is at 0.7, Figure 1a, c). Of the 15 sites, 11 are exposed to the surface (Figure S1A), 3 are positively charged amino acids (R35, K215, and R251, Figure 1d, e), and 2 are aromatic (Y30 and F219, Figure 1d, e), which are typical DNA‐binding amino acids (Anashkina, 2023). K215, F219, and R251 are located on neighboring *β*‐strands (Figure 1d). This region in the *β*‐barrel is an intriguing discovery due to the close proximity to two highly conserved C residues (C178 and C222 with conservation scores of 0.754 and 0.763, respectively), which are predicted to participate in the zinc‐binding site (Leipart et al., 2022). Also, the glycosylated N296 is in the last *β*‐strand in the barrel (Figure 1e), which positions the modification in the middle of the potential DNA‐binding sites.

### 3.2 Consistent structural alignments

**FIGURE 2:**
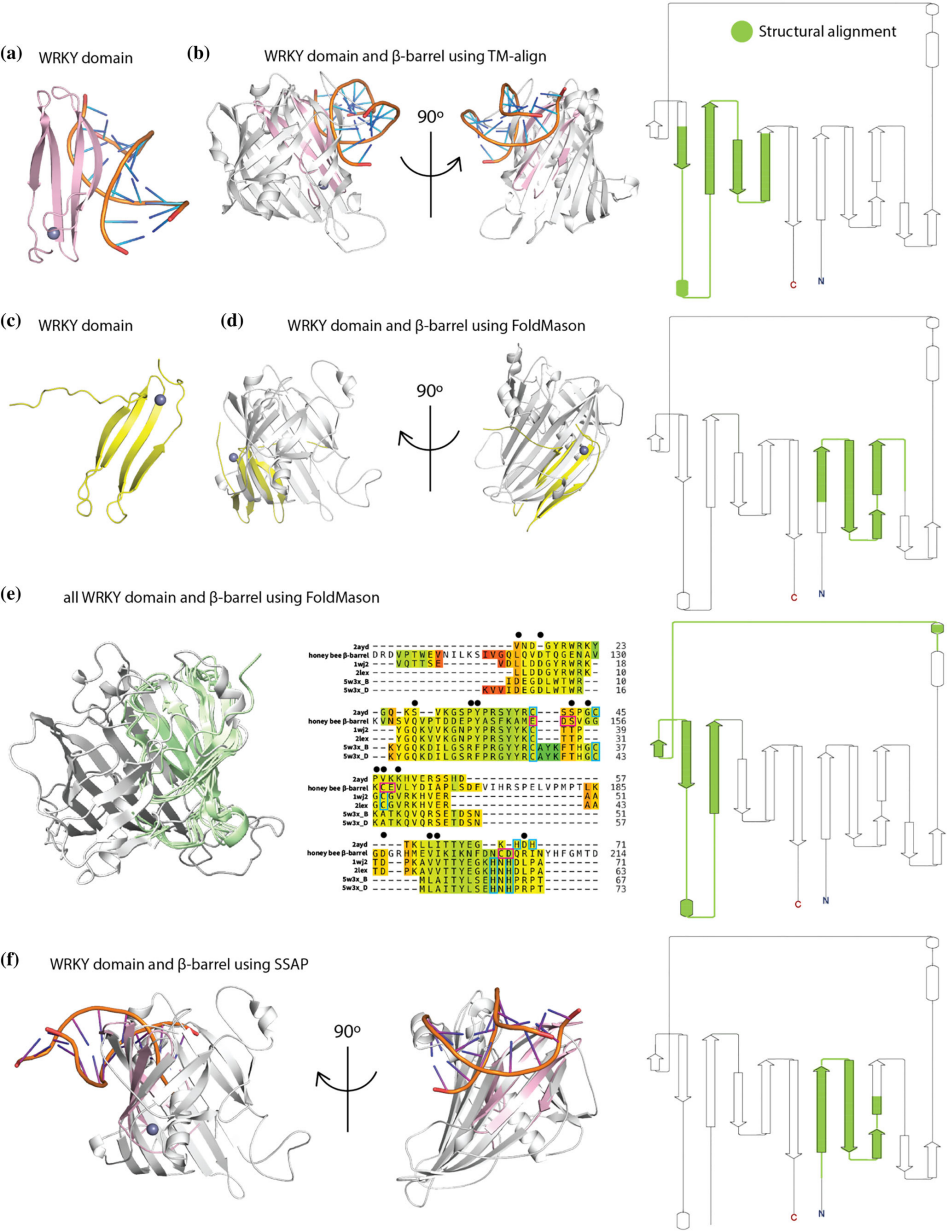
(a) The 3D structure of the WRKY domain (PDB ID 2LEX) includes a DNA strand (orange) and a zinc ion (gray). (b) Structural alignment of WRKY domain (pink) to the honey bee *β*‐barrel (white) from two directions, using TM align. To the right is a 2D map showing the overlapping regions in green. (c) The 3D structure of the WRKY domain (PDB ID 1WJ2), including a zinc ion (gray). (d) Structural alignment of the WRKY domain (yellow) to the honey bee *β*‐barrel (white) from two directions, using FoldMason. The 2D map shows the overlapping regions in green. (e) Multiple structural alignment using FoldMason to all the WRKY domains (honey bee *β*‐barrel in white, while all the WRKY domains are green), and a multiple sequence alignment colored by LDDT (red to green, red is low confidence and green is high confidence). Black dots illustrated were amino acids in the honey bee *β*‐barrel is conserved across the WRKY domains. Pink boxes are the putative zinc‐binding sites in Vg, while the blue boxes are the C_2_H_2_ zinc‐binding sites in the WRKY domain. The 2D map shows the overlapping regions in green. (f) Structural alignment of the top hit WRKY domain (PDB ID 2LEX, same domain as in panel a) to the honey bee *β*‐barrel (white) from two directions using SSAP. The 2D map shows the overlapping regions in green.

We performed pairwise and multiple structural alignments between all available structures of β‐sheeted DNA‐binding proteins, including a zinc‐binding site, to the honey bee *β*‐barrel structure. We used three different tools: TM‐align, FoldMason, and SSAP (Gilchrist et al., 2024; Orengo & Taylor, 1996; Zhang & Skolnick, 2005). TM‐align predicted that the *β*‐barrel and the WRKY domain have a similar fold (TM‐score >0.5 and RMSD 3.01–3.25 Å, Table S1) despite a low sequence identity (7%–10%). Alignment of the top hit from TM‐align (Figure 2a) aligned a WRKY domain to four *β*‐strands in the honey bee *β*‐barrel that includes K215, F219, and R251 (amino acids 180–278) (Figure 2b). FoldMason also found the highest confidence score (LDDT, see “Methods” for details) for a WRKY domain (Table S2, Figure 2c). Alignment of the top hit from FoldMason predicted an alignment of the WRKY domain to the four *β*‐strands in the N‐terminal region on the *β*‐barrel (amino acids 31–100) (Figure 2d). FoldMason also allows for multiple structure alignment, so we aligned all the WRKY domains available in the PDB to the honey bee *β*‐barrel, which resulted in an increased LDDT score and a similar alignment to the top hit from TM‐align (amino acids 139–227) (Figure 2e). The multiple alignment also identified patches of sequence conservation, which show that three putative zinc‐binding amino acids in the *β*‐barrel (E171, C178, and D223) align to the C_2_H_2_ zinc‐binding site of the WRKY domain (Figures 2e and S1B). The third pairwise structural alignment tool, SSAP, also found the highest SSAP score and lowest RMSD for the alignments to the WRKY domains (Table S3). Alignment of the top hit from SSAP aligned the WRKY domain to the N‐terminal region of the *β*‐barrel (amino acids 24–76), similar to the pairwise alignment by FoldMason (Figure 2f). Taken together, the structural alignments show that the honey bee *β*‐barrel has a significant structural similarity to the WRKY domain in two regions: the N‐terminal region from amino acids 23–100 and the C‐terminal region from amino acids 139–278. These regions overlap with our sequence analysis's predicted DNA binding sites.

### 3.3 Vg‐DNA binding

**FIGURE 3:**
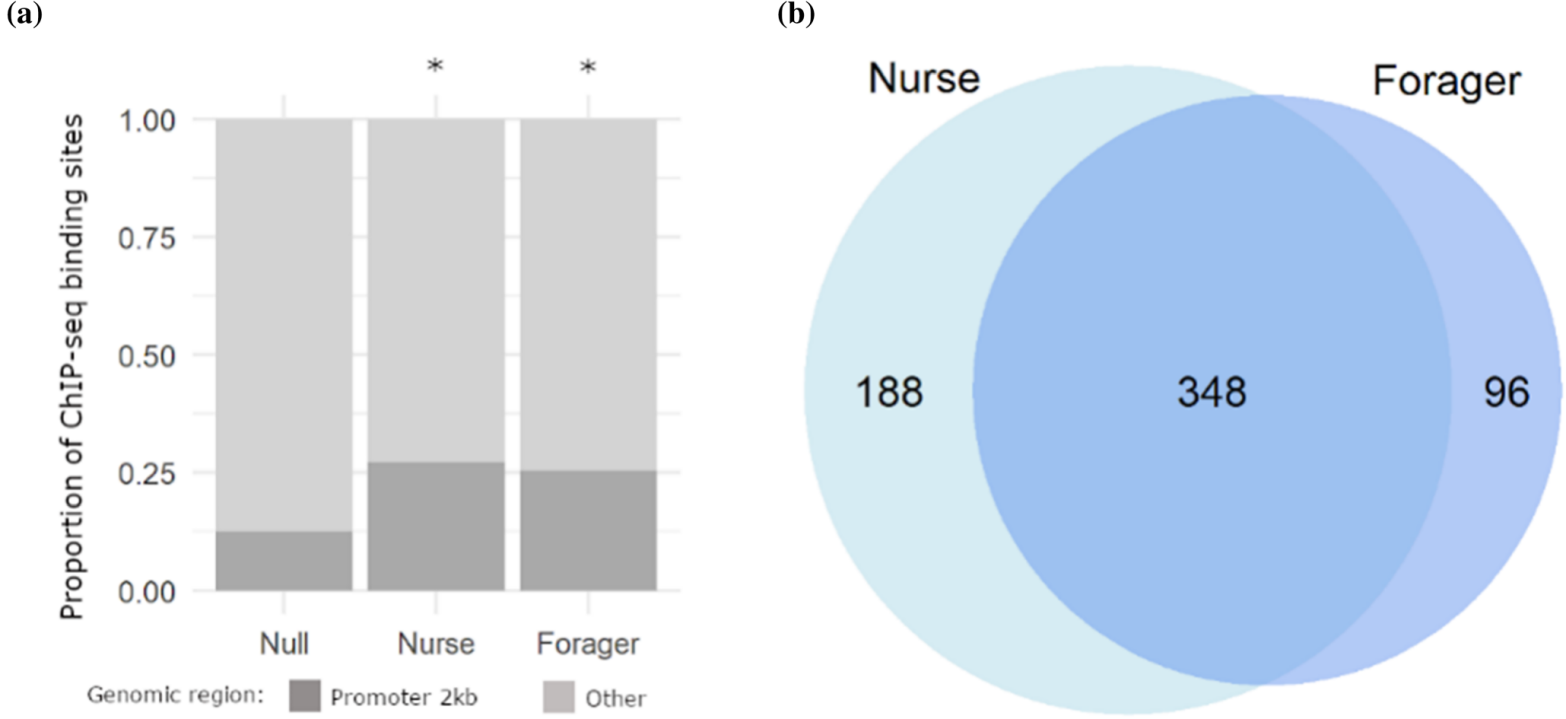
Results from ChIP‐seq analyses. (a) The distribution of ChIP‐seq binding sites in promoter regions compared to other genomic regions. A promoter region is defined as a window 1 kb upstream to 100 bp downstream from a transcription start site. Both nurses and foragers had significantly more sites in promoter regions compared to a null distribution of randomized ChIP‐seq peaks, as determined by χ^2^ Goodness of Fit tests. (b) ChIP‐seq sites were annotated to 596 separate genes. Nurse and forager samples shared peaks in a majority of these genes, while some were restricted to only nurses or foragers.

We found an average of 944 Vg‐DNA binding sites in nurses (range: 901–1020) and 930 in foragers (range: 867–1049) that were aligned to the honey bee genome. These binding sites were found in promotor regions with a higher probability than chance, with an average of 25% and 27%, respectively, found within a 1 kb window upstream of a TSS compared to 12.5% of sites observed here in a null distribution of randomly generated ChIP peaks (χ^2^ = 193.92, df = 1, *p* <0.001, χ^2^ = 137.47, df = 1, *p*<0.001) (Figure 3a) (Salmela et al., 2022). We trimmed away redundant binding sites annotated to the same gene and only considered genes that were shared by at least 2 of the 3 biological replicates per caste, leaving a total of 596 genes, hereafter referred to as *ChIP genes* for clarity. Of these ChIP genes, the majority were shared between nurses and foragers, while 188 and 96 were found only in nurses or foragers, respectively (Figure 3b). A GO term analysis did not find significant enrichment at the BH‐adjusted *p*‐value level for any biological processes, molecular functions, or known pathways, but there was enrichment at the unadjusted *p*‐value level for “signaling receptor activity” in nurses (GO: 0038023, unadjusted *p* = 0.024) and “methyltransferase activity” in foragers (GO: 0008168, unadjusted *p* <0.001).

### 3.4 Differential gene expression

**FIGURE 4:**
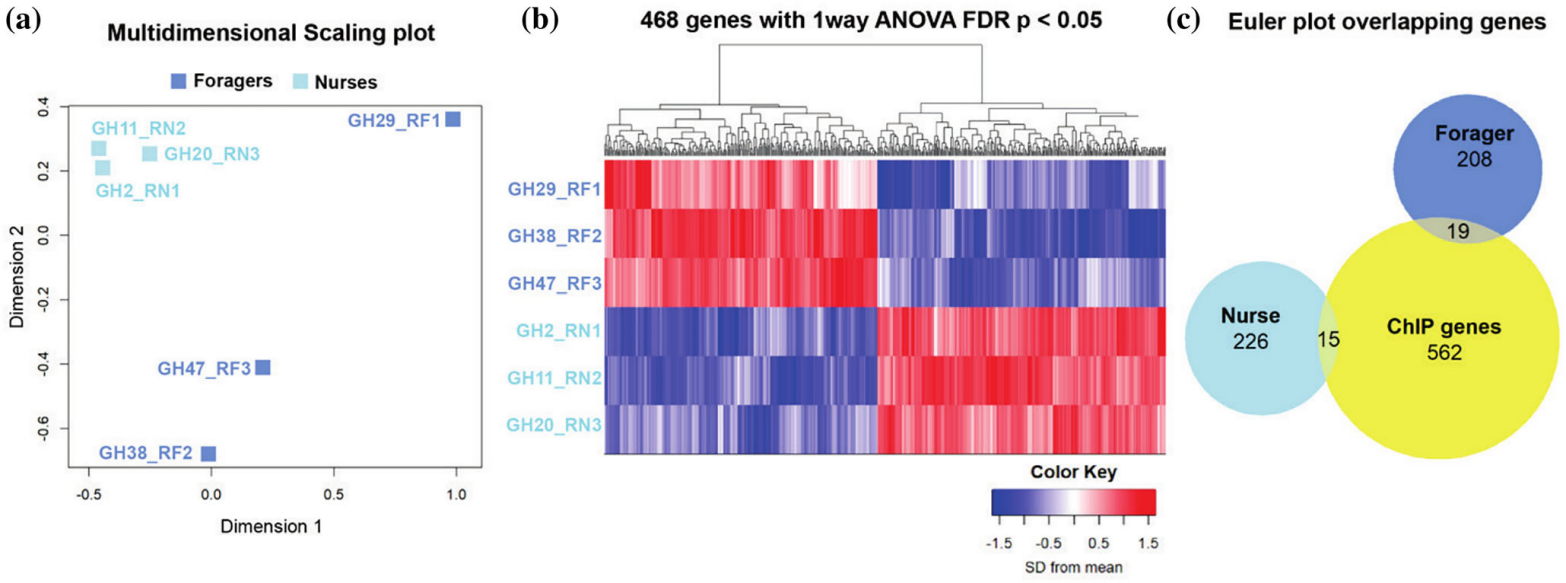
RNA‐seq expression results from nurses and foragers, and their overlap with ChIP‐seq results. (a) Multidimensional scaling of nurse and forager samples comparing variance of logCPM values for the 5000 most variable genes. The first dimension explains 47% of the variance and separates nurses from foragers, as well as accounts for some variance between forager samples. The second dimension explains 25% of the variance and accounts for additional variance among forager samples. (b) Heat map of genes that were differentially expressed (DEGs) between nurses and foragers (FDR <0.05). (c) Euler plot depicting the overlap in DEGs upregulated in nurses or foragers with genes identified as Vg binding targets via ChIP‐seq. Approximately 7% of genes bound by Vg show differential expression between nurses and foragers.

RNA‐seq analysis generated 41–62 million mappable reads per sample, detecting 9134 genes above a threshold cutoff of 0.5 CPM. Variation between castes and hives was examined using multidimensional scaling of logCPM values of the 5000 most variable genes. All nurse samples were found to be similar to each other and clustered closely together, while forager samples showed higher inter‐colony variation (Figure 4a). The cause of this increased variation among foragers is unknown but may be due to viral infection, as foragers had a higher proportion of their counts map to two known viral genomes. Differential expression testing yielded 468 DEGs between nurses and foragers using a one‐way ANOVA with an FDR *p* <0.05, of which 342 genes had a log_2_ fold change greater than 1 (Figure 4b). As expected, Vg was one of the most differentially expressed, showing levels nearly 8× higher in nurses than foragers (log_2_FC 2.97, BH‐adj. *p* = 0.009). GO term analysis of all DEGs showed enrichment for several biological processes, molecular functions, and KEGG pathways, including “oxidation–reduction process” (GO:0055114, BH‐adj. *p* <0.001), “catalytic activity” (GO:0003824, BH‐adj. *p* <0.001), and many metabolic pathways (Table 1).

**TABLE 1:** GO term enrichment for ChIP‐seq and RNA‐seq identified hits.

Description	Term ID	Adj. *p*‐value	Number of genes or proteins
RNA‐seq (all DEGs)			
Oxidation–reduction process	GO:0055114	2.26E‐13	57
Aromatic amino acid family metabolic process	GO:0009072	4.68E‐02	5
Cofactor binding	GO:0048037	8.12E‐08	37
Iron ion binding	GO:0005506	1.95E‐05	16
Catalytic activity	GO:0003824	2.33E‐05	151
Oxidoreductase activity, acting on paired donors, with incorporation or reduction of molecular oxygen	GO:0016705	5.47E‐05	16
Coenzyme binding	GO:0050662	1.89E‐04	21
Monooxygenase activity	GO:0004497	6.48E‐04	12
Heme binding	GO:0020037	1.66E‐03	14
Tetrapyrrole binding	GO:0046906	1.68E‐03	14
Flavin adenine dinucleotide binding	GO:0050660	1.41E‐02	10
Oxidoreductase activity, acting on CH–OH group of donors	GO:0016614	1.97E‐02	10
Chitin binding	GO:0008061	3.04E‐02	9
Valine, leucine and isoleucine degradation	ame00280	6.25E‐05	9
Tryptophan metabolism	ame00380	2.05E‐04	7
Fatty acid degradation	ame00071	2.64E‐04	7
Phenylalanine metabolism	ame00360	1.05E‐03	4
Glycine, serine and threonine metabolism	ame00260	4.12E‐03	6
Beta‐Alanine metabolism	ame00410	5.99E‐03	5
Drug metabolism—other enzymes	ame00983	6.37E‐03	6
Fatty acid elongation	ame00062	9.26E‐03	4
Glyoxylate and dicarboxylate metabolism	ame00630	1.04E‐02	5
Tyrosine metabolism	ame00350	1.12E‐02	4
Pyruvate metabolism	ame00620	3.66E‐02	5
Lysine degradation	ame00310	4.41E‐02	5
Overlapping ChIP‐seq sites and DEGs			
Heparan sulfate sulfotransferase activity	GO:0034483	5.42E‐03	2

### 3.5 Overlapping ChIP genes and DEGs

**FIGURE 5:**
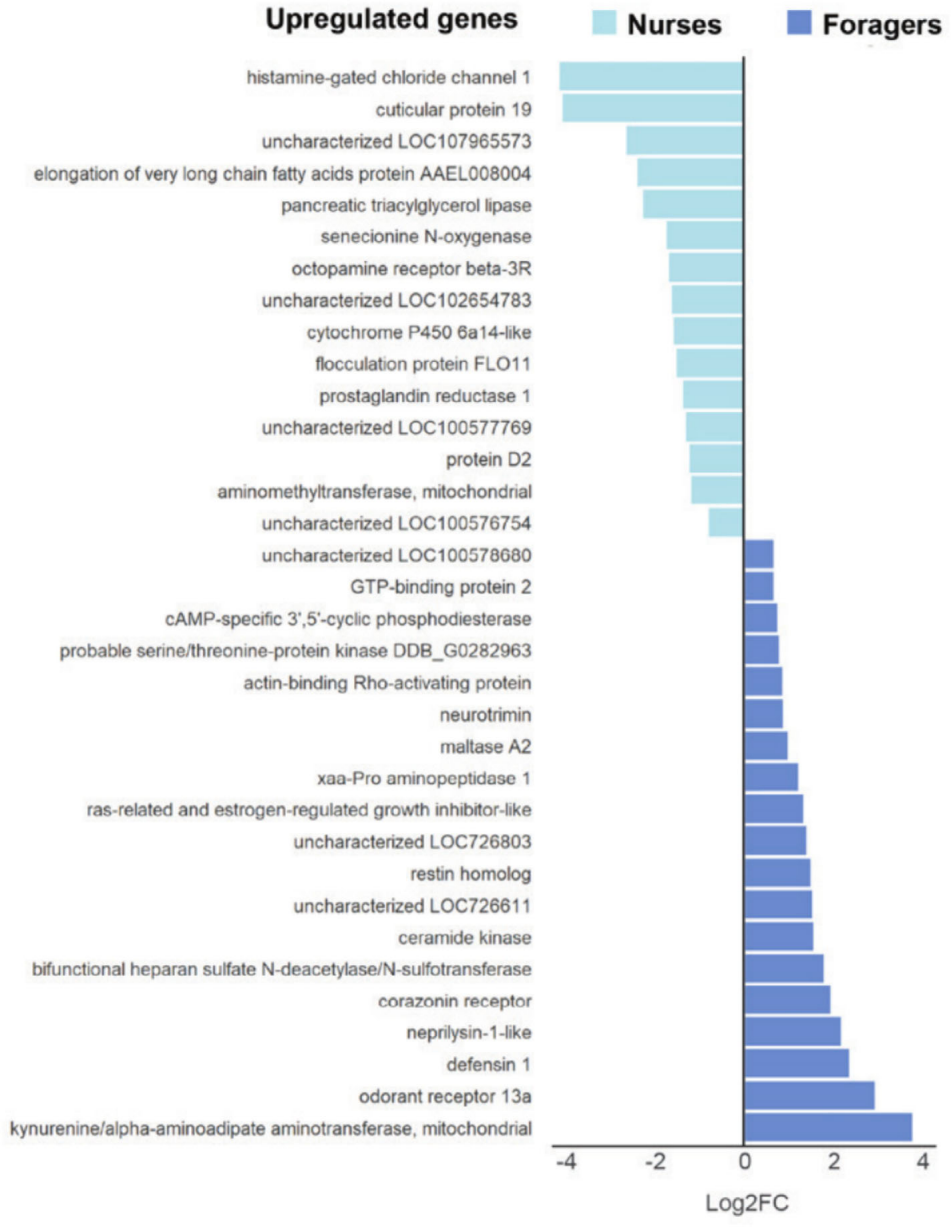
Candidate genes for Vg transcriptional regulation. These are ChIP‐genes that show differential expression between nurses and foragers (FDR <0.05). The *x*‐axis shows the log_2_ fold change, with nurse‐upregulated genes to the left and forager‐upregulated genes to the right.

We found that 34 genes bound by Vg also showed differential expression (Figure 4c), indicating these as candidate genes for direct transcriptional regulation by Vg (Figure 5a). This overlap of ChIP genes and DEGs represents ~7% of all Vg‐DNA binding sites, which is in line with other studies of transcription factors (Elsik et al., 2016; Guo et al., 2009; Mellacheruvu et al., 2013). Overlapping ChIP genes and DEGs showed enrichment for the GO term “heparan sulfate sulfotransferase activity” (GO:0034483, BH‐adj. *p* = 0.005) (Table 1). Heparan sulfate is a polysaccharide that binds to a variety of proteins and performs many functions pertaining to signal transduction, genomic regulation, and viral infection (Christianson & Belting, 2014; Pfeiffer et al., 2014; Pilon et al., 2011; Zhan et al., 2018). This term was also enriched when only considering genes upregulated in foragers, but there were no enriched terms for genes upregulated in nurses only. The Vg‐bound genes that show differential expression in nurses or foragers are involved in a broad set of biological functions. These include several receptors with known effects on behavior and signaling, a number of enzymes involved in oxidation–reduction pathways, and an important antimicrobial peptide used to fight infection (*Defensin‐1*).

### 3.6 Mass spectrometry of Vg‐bound nuclear proteins

**FIGURE 6:**
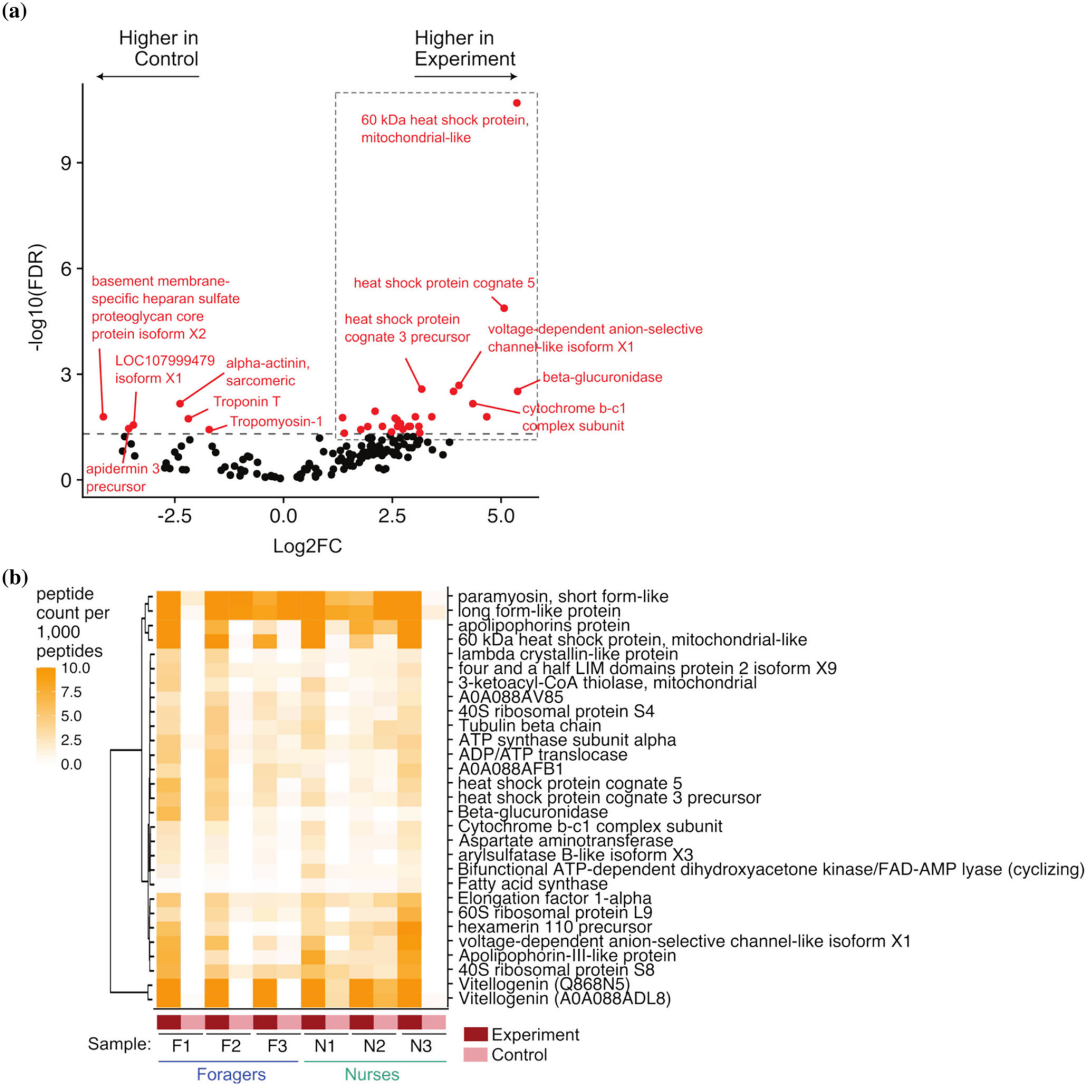
Mass spectrometry analysis of proteins bound to Vg‐DNA complexes. (a) Differential abundance analysis of experiment (i.e., anti‐Vg+) and control precipitations. Proteins with significant (false discovery rate [FDR] <0.05) difference between the two conditions are highlighted in red. Proteins significantly enriched in the treatment condition are highlighted with a gray box. (B) Heatmap depicting abundance levels for proteins significantly enriched in the treatment condition (see panel a). Abundance levels for each protein were represented by the peptide counts mapped to the protein normalized to a library size of 1000 peptides, to account for differences in the numbers of proteins identified in the mass spectrometry analysis. Proteins with unannotated function were represented with their UniProt identifiers.

Our co‐immunoprecipitation assay identified a total of 35 Vg‐bound nuclear proteins that were significantly (FDR <0.05) more abundant in comparison to our control precipitations (Figure 6a). The protein exhibiting the largest fold change was *β*‐glucuronidase, an enzyme involved in the hydrolysis of heparan sulfate that is known to co‐localize with heparan sulfate in the nucleus and be enzymatically active there (Zhan et al., 2018). Other Vg‐bound proteins identified in our analysis (Figure 6b) include apolipophorins that are known to be involved in lipid transport (Weers & Ryan, 2006) and innate immunity (Kim & Jin, 2015), and proteins involved in metabolism, such as 3‐ketoacyl‐CoA thiolase, ADP/ATP translocase, fatty acid synthase. In total, we identified 7 mitochondrial proteins bound to Vg in the nucleus, two of which are directly involved in oxidation to reduction processes. Additionally, we found heat shock proteins, which are activated during stress in honey bees, such as during viral infections (Shih et al., 2021).

## 4 DISCUSSION

It is established that Vg levels have fundamental impacts on behavior, physiology, and gene expression in honey bees (Amdam et al., 2005; Amdam, Norberg, et al., 2003; Amdam & Omholt, 2003; Ihle et al., 2010; Ihle et al., 2015; Salmela et al., 2016; Salmela et al., 2022; Wheeler et al., 2013), and that Vg and its receptor can influence complex traits in additional insects such as the mosquito *Aedes albopictus* (Dittmer et al., 2019) and subsocial beetle *Nicrophorus vespilloides* (Roy‐Zokan et al., 2015). Explanations for these profound effects have so far focused on regulatory relationships associated with female reproduction in which Vg integrates with hormones and/or nutrient‐sensing pathways (Amdam, Norberg, et al., 2003; Amdam & Omholt, 2003; Ihle et al., 2010; Münch & Amdam, 2010; Wheeler et al., 2013). In solitary species, this integration ensures that egg production and associated female behaviors such as host seeking or nest construction are coordinated (Amdam & Page, 2010; Dittmer et al., 2019; Splitt et al., 2022). In ancestors of social species, the same integration was a pre‐adaptation for the evolution of female insect castes that differ in reproductive capabilities and behavior (Du et al., 2017; Zhang et al., 2015; Zhao et al., 2023). We propose that Vg, in addition to such interplay with hormones and sensing pathways, can influence genes and their phenotypes via direct binding to DNA.

In this study, we assess Vg's potential for DNA binding and gene regulation in honey bees. The region we zoomed in on is the N‐terminal β‐barrel, which is conserved for all members in the Large Lipid Transfer Protein superfamily, including mammalian apolipoprotein B (ApoB) and MTP (Baker, 1988b; Doublet et al., 2017). Intriguingly, a question of DNA‐binding capability was explored for ApoB over 10 years ago, with one candidate region located in the *β*‐barrel (Guevara et al., 2010). This former research focused on ApoB binding to viral DNA with implications for animal transfection experiments, and the team could show that DNA bound to LDL molecules (low density lipoprotein what has ApoB as its major protein) was transferred to the cell nucleus. Similarly, an engineered molecule with structural features of the Vg *β*‐barrel was recently leveraged for access to the nucleus to deliver Cas9/sgRNA complexes in the eggs of the sea star *Patiria miniata* (Clarke et al., 2024). In contrast to these efforts, we ask whether the natural Vg *β*‐barrel is likely to bind nuclear DNA to regulate genes, and we use intact, adult honey bee workers as a study system.

Our investigation identified conserved DNA‐binding amino acids in the Vg *β*‐barrel, in structural regions similar to *β*‐sheeted DNA‐binding proteins. Our analysis suggests two potential binding sites, located at the N‐ and C‐terminal. The top pairwise structural alignments from FoldMason and SSAP align the WRKY domain to the N‐terminal region of the *β*‐barrel (amino acid 24–100). In this sequence stretch we find typical DNA‐binding amino acids conserved (Y30 and R35) and the *β*‐strands are supported by the central *α*‐helix in the domain and possibly by a putative zinc‐binding site, although none of the zinc‐binding amino acids (H20, H113, D143, E147, and H265 (Leipart et al., 2022)) is included in the structural alignment. Moreover, the C‐terminal includes the SRSSTSR motif (amino acid 250–256), which we previously speculated could be involved in DNA binding (Salmela et al., 2022) and have the most surface‐exposed DNA‐binding amino acids (Figure S1A). Here, we find that our most confident structural alignment (RMSD of 3.23 Å and TM‐score of 0.59) supports the DNA binding through the C‐terminal. The structural alignment also overlaps with conserved DNA‐binding amino acids, and in addition includes zinc‐binding amino acids. Similarly to the N‐terminal, the central *α*‐helix would also support the C‐terminal DNA binding site.

Both sites have the highest structural similarity to the WRKY domain, and we also identify patches of sequence identity (14%–10%) between the WRKY domain and the *β*‐barrel. The low sequence identity combined with the fact that the WRKY domain is only found in plants makes us find it unlikely that Vg and WRKY are evolutionarily related. Instead, we propose that Vg has adopted a similar structural solution for DNA binding through structural convergence. We show that putative zinc‐binding amino acids at the C‐terminal DNA site in the *β*‐barrel align with the C_2_H_2_ zinc‐binding site in the WRKY domain, although the specific amino acid type is only conserved at one position (C178) (see structural alignment in Figure S1B) and the specific WRKY motif (WRKY sequence in Figure 2e) is not conserved. This is expected as the two proteins evolved in different organisms that likely have different DNA targets. This might explain why we observe minor clashes during the superimposition of the DNA strand from the WRKY structures with the *β*‐barrel domain (Figure 2b, f), as the DNA strand in the honey bee might differ. Conservation of the C_2_H_2_ site across *β*‐sheeted DNA binding domains is suggested to indicate similar DNA interaction mechanisms (Babu et al., 2006). Therefore, we suggest that Vg has convergently evolved to use outward‐facing *β*‐strands positioned perpendicular in the major groove of the DNA helix, supported by zinc. We find it likely that both DNA binding sites in the *β*‐barrel can additionally be supported by the central *α*‐helix, as seen for other *β*‐sheeted DNA binding domains (Allen et al., 1998; Campagne et al., 2010; Wojciak et al., 1999) and glycosylation, as seen for other DNA‐binding proteins (Kim et al., 2016). Vg is glycosylated in the center of the *β*‐barrel, potentially facilitating interactions at both the N‐ and C‐terminals. We also demonstrate that conserved DNA‐binding amino acids are present in human MTP and ApoB proteins (see Supplement Figure S2), indicating that the DNA‐binding functionality is likely conserved across the superfamily.

Further studies on Vg's binding mechanism could help describe the specific DNA binding mechanism and what structural elements, metal ions, and modifications support the binding. Our structural analysis uses a static model of the honey bee domain, which may not accurately reflect the true binding interfaces. We have recently demonstrated that the domain contains two highly dynamic loops near the C‐terminal region ((Leipart et al., 2025) and Figure S1). Their static fold in our model may shield DNA‐binding interfaces. These dynamics, and potential participation in DNA binding, can be captured through molecular dynamics (MD) simulations, for example. We propose that full MD simulations, including the *β*‐barrel domain with DNA, glycosylation, and zinc, would provide valuable insight into how the domain binds to DNA.

While our study reveals that Vg has two potential sites for DNA binding, a question that remains is whether they act independently or together. An intriguing scenario to explain the numerous regulatory and transcriptional events found here is that Vg can bind to two distinct DNA motifs utilizing the sites independently. Potentially, each site would then lead to different regulatory or transcriptional events. Another scenario is that Vg binds to DNA using both sites simultaneously. This binding mechanism could be possible through homodimer formation, as described for the *β*‐sheeted Arc repressor (Raumann et al., 1994).

In terms of the functional aspects of Vg‐DNA binding, we found that Vg binds to over 600 genes and is more likely than chance to be found in promoter regions, where transcription factors typically bind (Cozzi et al., 2003). We also found that Vg‐DNA binding is associated with differential gene expression in 34 genes of nurses and foragers, and that 35 additional nuclear proteins bind to the Vg‐DNA complex. The number of genes that Vg appears to bind to, and how many of these are differentially regulated, falls within ranges previously established for transcription factors, such as forkhead box (FOX) transcription factors (Li et al., 2015; Singh et al., 2018). In addition, the number of additional nuclear proteins that we found associated with Vg is also an expected outcome based on similar studies where FOX transcription factors were co‐immunoprecipitated with nuclear proteins (Li et al., 2015; Singh et al., 2018) Furthermore, we find unique ChIP genes for nurses and foragers, which could result from several regulating mechanisms affecting Vg‐DNA binding. One recently discovered possibility is epigenetic markers. For example, DNA methyltransferase 3 has been shown to regulate worker behavior maturation, possibly through affecting Vg expression (Cardoso‐Júnior et al., 2018).

In terms of the potential target genes of Vg, our data can be leveraged to suggest possible biological processes under Vg control, some of which may contribute to differences observed between honey bee nurses and foragers. These include energy metabolism and subsequent reactive oxygen species production, behavior, and signaling. Specifically, both the list of all DEGs between nurses and foragers and the list of nuclear proteins bound to the Vg‐DNA complex are most highly enriched for the term “oxidation–reduction process” Redox reactions are enzymatic reactions used to transfer electrons between chemicals and are often associated with cellular respiration in mitochondria, where nutrients are oxidized in the production of ATP. The behavioral change from nursing to foraging is accompanied by massive changes in metabolic biology as foraging requires flight and a shift to a sugar‐based diet for fuel, so altered redox gene expression is expected (as also observed by (Pham et al., 2004)). However, our additional finding of similarly categorized proteins in the Vg‐DNA complex might indicate that enzymes used in redox reactions interact with Vg or Vg‐associated proteins to influence gene expression. Intriguingly, two of the redox proteins we found bound to the Vg‐DNA complex are also mitochondrial proteins. Mitochondria‐to‐nucleus signaling can target nuclear genes to reconfigure metabolic pathways (Gill, 2001; Segrest et al., 1999) and reduce oxidative stress (Azzouz‐Olden et al., 2018), among other functions. While reactive oxygen species may be best known for causing cellular damage, they can also activate transcription factors like NF‐κB that upregulate antioxidant and DNA‐repair genes (Khan et al., 2018; Smith et al., 2018). Thus, we speculate that our data hint at a relationship between Vg and redox enzymes in which Vg plays an evolutionarily conserved role in mitochondria‐to‐nucleus signaling that allows females to coordinate energy expenditure and somatic maintenance with the demands of reproduction. If so, Vg would take on a role similar to STAT3, which can localize in mitochondria and perform oxidative phosphorylation functions and also translocate to the nucleus as a transcription factor to regulate many processes like cell proliferation and apoptosis (Jazwinski, 2015).

Our data, furthermore, show that Vg‐DNA binding is associated with expression changes in several behavior‐related genes. *Histamine‐gated chloride channel* 1, which is highly upregulated in nurses, can synchronize activity during light–dark cycles in *Drosophila* (Storz, 2006), while *odorant receptor 13a* may play a role in brood pheromone perception in honey bees (Storz et al., 2005). But perhaps more interestingly, foragers upregulated expression of the corazonin receptor: Corazonin is a neuropeptide that, in a distant relative of the honey bee, the *Harpegnathos* ants, has a co‐repressive regulatory relationship with Vg to control caste identity and behavior (Wegrzyn et al., 2009). In this ant, high levels of Vg in nurses repress corazonin receptor expression via an unknown mechanism, while reduction of Vg in foragers (naturally as well as experimentally) appears to release or promote expression of the receptor. We speculate that the unknown mechanism responsible for this dynamic regulation is Vg‐DNA binding.

Another example of patterns in our data is that there is significant enrichment for heparan sulfotransferase activity in the list of overlapping ChIP genes and DEGs, and the most strongly enriched protein in our proteomics analysis of anti‐Vg precipitations, *β*‐glucuronidase, is an enzyme that interacts with heparan sulfate in the nucleus (Zhan et al., 2018). Heparan sulfate often binds with proteins to form heparan sulfate proteoglycans (HSPGs), which are active on the plasma membrane and act as receptors for many ligands (Alejevski et al., 2019). It is also of note that heparan sulfate in the nucleus is believed to play several roles in regulating gene expression and proliferation, as well as transport of molecules into the nucleus (reviewed in (Christianson & Belting, 2014)). Indeed, a relationship between Vg‐like proteins and HSPGs has been documented in *Drosophila*: here, *crossveinless d* (*csv‐d*) codes a Vg homolog that contains a *β*‐barrel structure—and this barrel binds to HSPGs to play a role in the bone morphogenic protein (BMP) signaling pathway (Oxley et al., 2008). These previous findings could indicate that Vg plays multiple roles in the cell nucleus, and not all may require direct DNA binding.

In this study, we choose to work with intact honey bee workers of different castes to learn more about how Vg may interact with DNA and what consequences such interaction might have. This design leads to potential shortcomings in that we lack a comparison between Vg‐wildtype bees and Vg‐knockout or ‐knockdown individuals, as is a common approach in studies of how transcription factors affect expression. However, unfortunately, honey bees have a very limited toolkit for knockout experiments (Chen et al., 2012; Gospocic et al., 2017; Sarrazin et al., 2011) and still have challenges to overcome regarding efficacy and the effects of rearing larvae in vitro (Hu et al., 2019). Moreover, even if the technology was sufficient, Vg‐knockout is likely lethal or with considerable health impacts given the key role Vg plays in development and immunity and the fact that honey bees only have one Vg gene (Salmela et al., 2016; Zhao et al., 2021). The first Vg‐knockout to date used the vertebrate model *Danio rerio*, which has several Vg genes, and still showed that offspring of Vg‐knockout parents displayed several lethal phenotypes (Kohno et al., 2016). The alternative approach, RNAi‐mediated Vg knockdown, is a method our team established more than 20 years ago (Amdam, Simões, et al., 2003) and that we have used extensively in research (Du et al., 2019; Guidugli et al., 2005; Ihle et al., 2015; Marco Antonio et al., 2008; Nelson et al., 2007; Schlüns & Crozier, 2007). This approach, unfortunately, is also problematic as *vg* gene knockdown causes workers to become precocious foragers (De Souza et al., 2018; Roth et al., 2019). This implies that the contrast between Vg‐wildtype and Vg‐knockdown workers is essentially the same as the same‐aged nurse and forager contrast we have achieved here using single‐cohort colonies. In support of this, our same‐age workers' nurses *vg* expression was ~8 times higher than in foragers, which is a similar degree of difference observed in Vg RNAi studies (Wheeler et al., 2013; Yilmaz et al., 2019). In this context, the single‐cohort approach is superior as it reduces the burden of experimental handling and treatment on the bees, leading to better animal welfare and reduced risks of data that reflect some level of stress‐induced responses.

With our specific explanation for how the Vg *β*‐barrel domain can bind DNA and act as a possible transcription factor, we help provide a mechanistic understanding of how Vg affects many traits. Indeed, the extent of Vg's multifunctionality is impressive (Amdam, Norberg, et al., 2003; Harwood et al., 2019; Havukainen et al., 2013; Salmela et al., 2015; Salmela et al., 2022; Seehuus et al., 2006). For perspective, it is important to realize that Vg may be just one of many molecules that can potentially regulate gene expression, as over 600 transcriptional regulators were estimated in the honey bee genome based solely on the presence of known DNA‐binding domains (Huang et al., 2012). Our current study determines that Vg has several sequence and structural features that should allow direct DNA binding. We also shed light on how Vg might interact with other molecules in the nucleus, as well as its potential gene regulatory pathways. While many questions remain, including what signals prompt Vg nuclear translocation and how Vg crosses the nuclear envelope, the data presented here offer insight into the types of biological processes that Vg may influence. They also offer an opportunity for new avenues for Vg research that were previously unknown or overlooked, including possible signaling pathways involving Vg and the mitochondria. Given that the Vg *β*‐barrel domain is conserved across metazoan taxa, we hope that our findings can inspire research on whether and how Vg and Vg homologs may interact with DNA in a variety of organisms.

## AUTHOR CONTRIBUTIONS

**Gyan Harwood:** Conceptualization; data curation; formal analysis; investigation; methodology; project administration; software; supervision; validation; visualization; writing – original draft; writing – review and editing. **Vilde Leipart:** Data curation; formal analysis; investigation; methodology; software; validation; visualization; writing – original draft; writing – review and editing. **Chris Elsik:** Formal analysis; investigation; methodology. **Joseph C. F. Ng:** Formal analysis; software; methodology; validation; visualization; writing – review and editing. **Finn Drabløs:** Investigation. **Gro V. Amdam:** Conceptualization; funding acquisition; project administration; resources; supervision; validation; writing – review and editing.

## CONFLICT OF INTEREST STATEMENT

The authors declare that they have no conflicts of interest with the contents of this article.

## Supporting information


**Figure S1:** shows the surface‐exposed DNA amino acids and structural alignment of zinc binding sites.

**Figure S2:** shows conserved putative DNA‐binding amino acids in MTP and ApoB.

**Data S1:** Chip_RNAseq_Proteins_GO: Contains the ChIP sites, RNAseq DEG analysis, list of nuclear proteins and the GO term analyses for these datasets.

**Data S2:** promoter region binding: Contains the test for promotor region binding.

**Tables S1–S3:** contains the output scores from TM‐align, FoldMason and SSAP, respectively.

## Data Availability

The data that supports the findings of this study are available in the supplementary material of this article.
