# Supplementary material for: Evidence for vitellogenin DNA‐binding in honey bees
Source: Protein Sci. 2025 Sep 13;34(10):e70291. doi: 10.1002/pro.70291 (PMC12432429; doi:10.1002/pro.70291)
Supplement: Supplementary file 1 — Figure S1: shows the surface‐exposed DNA amino acids and structural alignment of zinc binding sites. [file PRO-34-e70291-s002.docx]

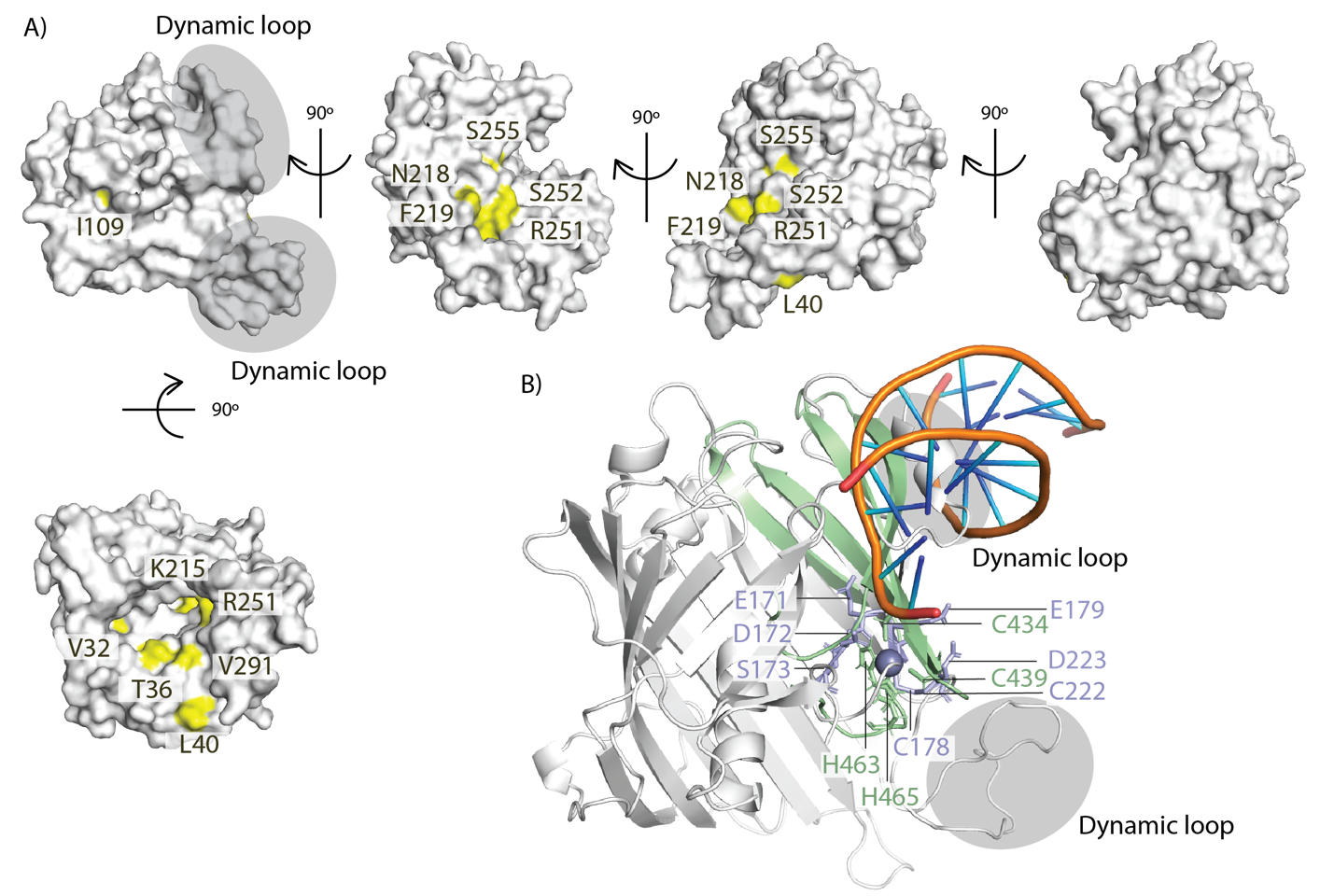


**Fig. S1 A:** The surface of the honey bee β-barrel (light grey) shown from four different angles turned around the y-axis from and one angle turned around the x-axis. The yellow colored surfaces are the putative DNA binding residues that are exposed to the surface. These are also labeled. In the first view we have highlighted with darker grey circles the two dynamic loops identified in the molecular dynamic simulations of the honey bee β-barrel domain. **B:** The 3D structure of the honey bee β-barrel (light grey) superimposed with 2LEX (green) using the multiple structural alignment by FoldMason. The DNA strand (orange) and Zn (dark grey) ion are from the 2LEX structure. Here we show that the zinc binding site in the WRKY domain is overlapping with the putative zinc binding site in honey bee β-barrel domain. The residues involved in the zinc binding are labeled in green (WRKY domain) or light blue (honey bee β-barrel). We have also highlighted the two dynamic loops with dark grey circles as in panel A.
