# Supplementary material for: Evidence for vitellogenin DNA‐binding in honey bees
Source: Protein Sci. 2025 Sep 13;34(10):e70291. doi: 10.1002/pro.70291 (PMC12432429; doi:10.1002/pro.70291)
Supplement: Supplementary file 2 — Figure S2: shows conserved putative DNA‐binding amino acids in MTP and ApoB. [file PRO-34-e70291-s004.docx]

**
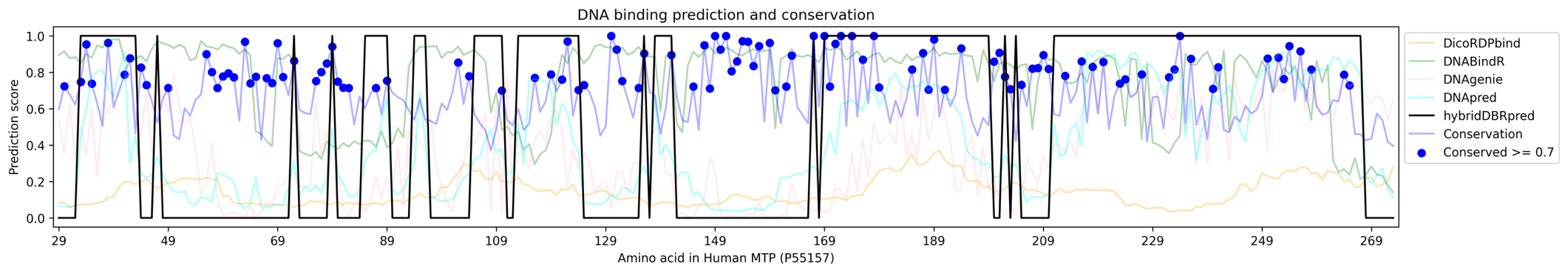
**

**
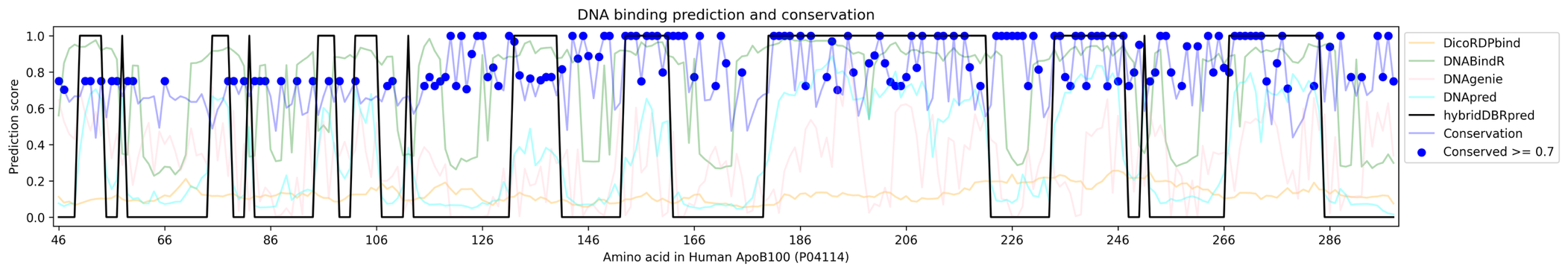
**

**Fig. S2** HybridDBRpred combines DNA prediction scores (y-axis) from 4 different tools (saturated yellow, green, pink, and cyan lines) that are combined into a binary DNA-binding prediction (1 yes or 0 no, black line) for all amino acids in the **A:** MTP and **B:** ApoB protein sequences (x-axis). In the same plot, we have included the conservation score (blue line) and labelled conserved amino acids >= 0.7 with spheres. These results show that there are conserved predicted DNA-binding amino acids in MTP and ApoB.
