# Supplementary material for: Evidence for vitellogenin DNA‐binding in honey bees
Source: Protein Sci. 2025 Sep 13;34(10):e70291. doi: 10.1002/pro.70291 (PMC12432429; doi:10.1002/pro.70291)
Supplement: Supplementary file 5 — Tables S1–S3: contains the output scores from TM‐align, FoldMason and SSAP, respectively. [file PRO-34-e70291-s005.docx]

| Protein Family | PDB ID, chain | RSMD | TM-score relative to target | Sequence Identity | Structural alignment to honey bee Vg |
| --- | --- | --- | --- | --- | --- |
| WRKY | 5w3x, B | 3.23 | 0.59 | 10 % | 180-278 |
|  | 5w3x, D | 3.25 | 0.59 | 10 % | 180-278 |
|  | 2ayd, A | 4.1 | 0.42 | 4 % | 210-320 |
|  | 2lex, A | 3.01 | 0.56 | 7 % | 180-278 |
|  | 1wj2, A | 3.95 | 0.46 | 5 % | 267-320 |
| GCM | 1odh, A | 5.21 | 0.35 | 5 % | 180-275 |
| THAP | 3kde, C | 4.06 | 0.32 | 2 % | 108-177 |
|  | 2jm3, A | 4.46 | 0.40 | 0 % | 140-297 |
|  | 2lau, A | 4.82 | 0.32 | 3 % | 137-296 |

**Structural alignments**

**Table S1:** TM-align structural alignments of honey bee β-barrel amino acids 20-323.

| Protein Family | PDB ID, chain | LDDT | Structural alignment to honey bee Vg |
| --- | --- | --- | --- |
| WRKY | 5w3x, B | 0.090 | 139-226 |
|  | 5w3x, D | 0.067 | 249-300 |
|  | 2ayd, A | 0.088 | 140-226 |
|  | 2lex, A | 0.067 | 24-77 |
|  | 1wj2, A | 0.100 | 31-100 |
|  | 5w3x B, 5w3x D, 2ayd, 2lex, 1wj2 | 0.145 | 139-227 |
| GCM | 1odh, A | 0.094 | 156-312 |
| THAP | 3kde, C | 0.070 | 170-247 |
|  | 2jm3, A | 0.073 | 139-274 |
|  | 2lau, A | 0.049 | 171-243 |

**Table S2:** FoldMason structural alignment of honey bee β-barrel amino acids 20-323.

| Protein Family | PDB ID, chain | SSAP score | Aligned amino acids | Overlap (%) | Sequence identity | RMSD | Structural alignment to honey bee Vg |
| --- | --- | --- | --- | --- | --- | --- | --- |
| WRKY | 5w3x, B | 47.49 | 48 | 15 | 14 | 5.71 | 24-76 |
|  | 5w3x, D | 47.12 | 48 | 15 | 13 | 5.24 | 24-76 |
|  | 2ayd, A | 47.36 | 46 | 15 | 7 | 4.45 | 24-75 |
|  | 2lex, A | 48.84 | 45 | 14 | 4 | 5.13 | 24-75 |
|  | 1wj2, A | 46.50 | 45 | 14 | 4 | 5.24 | 23-75 |
| GCM | 1odh, A | 49.71 | 97 | 32 | 1 | 9.11 | 136-278 |
| THAP | 3kde, C | 34.49 | 74 | 24 | 4 | 14.94 | 108-294 |
|  | 2jm3, A | 37.62 | 71 | 23 | 5 | 14.65 | 120-178 |
|  | 2lau, A | 30.12 | 65 | 21 | 4 | 12.18 | 127-298 |

**Table S3:** SSAP structural alignment of honey bee β-barrel amino acids 20-323.
